# Deletion of FaeG alleviated Enterotoxigenic *Escherichia coli* F4ac-induced apoptosis in the intestine

**DOI:** 10.1186/s13568-021-01201-z

**Published:** 2021-03-18

**Authors:** Pengpeng Xia, Yunping Wu, Siqi Lian, Guomei Quan, Yiting Wang, Guoqiang Zhu

**Affiliations:** 1 College of Veterinary Medicine (Institute of Comparative Medicine), Yangzhou University, Yangzhou 12th East Wenhui Road, Yangzhou, 225009 China; 2 Jiangsu Co-Innovation Center for Prevention and Control of Important Animal Infectious Diseases and Zoonoses, Yangzhou, 225009 China; 3 Joint International Research Laboratory of Agriculture and Agri-Product Safety of Ministry of Education of China, Yangzhou University, Yangzhou, 225009 China

**Keywords:** Enterotoxigenic *Escherichia coli* (ETEC) F4ac, FaeG subunit, Apoptosis

## Abstract

Enterotoxigenic *Escherichia coli* (ETEC) F4ac is a major constraint to the development of the pig industry, which is causing newborn and post-weaning piglets diarrhea. Previous studies proved that FaeG is the major fimbrial subunit of F4ac *E. coli* and efficient for bacterial adherence and receptor recognition. Here we show that the *faeG* deletion attenuates both the clinical symptoms of F4ac infection and the F4ac-induced intestinal mucosal damage in piglets. Antibody microarray analysis and the detection of mRNA expression using porcine neonatal jejunal IPEC-J2 cells also determined that the absence of FaeG subunit alleviated the F4ac promoted apoptosis in the intestinal epithelial cells. Thus, targeted depletion of FaeG is still beneficial for the prevention or treatment of F4ac infection.

**Supplementary Information:**

The online version contains supplementary material available at 10.1186/s13568-021-01201-z.

## Introduction

Enterotoxigenic *Escherichia coli* (ETEC) F4 is the leading cause of diarrhea in neonatal and post-weaning piglets (Sugiharto et al. 2012; Van den Broeck et al. 2002). The bacteria induce the disease depending on the interaction between fimbriae and host receptors, while fimbriae-mediated attachment to the intestinal epithelium is the initial step in these infections (Xia et al. 2015b). Among these three fimbrial variants, i.e., F4ab, F4ac, and F4ad, F4ac is the most common serotype and FaeG is the major subunit of F4 fimbriae (Sugiharto et al. 2012; Van den Broeck et al. 2002). Our previous study has shown that FaeG mediates the binding of F4ac *E. coli* with both porcine brush border cells and IPEC-J2 cells and interacts with the receptor of F4 fimbriae directly (Xia et al. 2016, 2015b).

Indeed, ETEC infections always increase intestinal epithelial permeability, villus atrophy, crypt hyperplasia and excessive cell apoptosis due to the release of toxins or other virulence factors (Tsai et al. 2017; Wang et al. 2011; Zhou et al. 2012). F4 *E. coli* promotes intestinal epithelial cell (IEC) apoptosis in piglets, which is mostly associated with the extrinsic pathway of apoptosis and leading to the activation of caspase-3 and caspase-8, resulting in intestinal barrier dysfunction and prolonged diarrhea (Xia et al. 2018b, 2019). The present study aims to investigate whether the FaeG subunit has a role in F4ac-induced cell apoptosis and the modulation of intestinal barrier function.

## Material and methods

### Bacterial strains, cell lines, and culture conditions

F4ac^+^ *E. coli* (G205 or C83902, O8:K87: F4ac) (Willemsen and de Graaf 1992) and the isogenic Δ*faeG* mutant (Xia et al. 2015a) were grown in Luria Bertani (LB) media (Solarbio, Beijing, China) with continuous agitation (178 rpm) at 37 °C. Porcine neonatal jejunal IPEC-J2 cells were grown in DMEM (Gibco, Australia) supplemented with 10% fetal bovine serum (FBS, Gibco, Australia) at 37 °C in a humidified incubator with an atmosphere of 6% CO_2_ (Xia et al. 2016).

### Animal infection experiment

Nine of 25-day-old Landrace and Large White 2-way crossbred Pigs were screened according to our previous studies (Xia et al. 2018a, 2015a). These piglets are susceptible to F4ac^+^ *E. coli* and randomized into three groups: the control group without ETEC infection, F4ac infected group and F4acΔ*faeG* infected group. Piglets were fed with 150,000 U/kg colistin for five days to clear intestinal flora. Before bacterial inoculation, the piglets from all groups were challenged orally with 30–60 mL 1.4% (w/w) NaHCO_3_ to neutralize gastric acid. After that, F4ac and F4acΔ*faeG* infected groups were fed with 3–5 mg/10 mL (5 × 10^9^ CFU/mL) bacteria every day for three consecutive days, while the control group was fed with 10 mL PBS at the same time. Bodyweight and temperature were measured once a day from the day before infection, and the development of disease in these piglets was observed and recorded during the infectious process. Piglets were sacrificed using CO_2_ gas after 5 days post infection.

### Histological observation

The harvested segments of the duodenum, jejunum, and ileum were flushed and fixed with 10% neutral-buffered formalin at room temperature for 24–48 h prior to the following preparation of paraffin block. The tissue was dehydrated and virtually transparent before infiltrated with paraffin wax. The paraffin-embedded tissue section is trimmed and frozen at – 20 ℃ for 24 h. The tissue wax was sliced by a rotary microtome and readily flatten in a warm water bath with a temperature of 45 ℃. 4 μm thick microtomed slices were mounted on microscope slides and dried on a hot plate at 60 °C for 3 h and then will be ready for staining. For Methylene Blue staining, the sample slides were flooded on 0.5% methylene blue (Solarbio, Beijing, China) for 2–3 min and then rinsed in distilled water to remove the excess stain before observing under a microscope. For Hematoxylin and eosin (H&E) staining, slides were deparaffinized and re-hydrated and then flushing with running water before stained the nucleus with hematoxylin (Solarbio, Beijing, China). After rinsing with water again, slides were quickly differentiated in 1% acid alcohol for 2 s and washed in tap water before counterstained with 0.5% ammonia (Solarbio, Beijing, China) for 1 min. Later, slides were stained in eosin solution for 30 s to 1 min and then washed and mounted before examining under the Olympus DP73 digital microscope (Areia et al. 2008; Fischer et al. 2008).

### Antibody microarray analysis

The Phospho Explorer Antibody Array kit used in this study was obtained from Full Moon BioSystems (catalog PEX100; Sunnyvale, CA, USA) and covered 1318 well-characterized antibodies, the phosphorylated and non-phosphorylated state of these proteins will be detected at the same time (Pulito et al. 2016). IPEC-J2 cells were incubated with 5 mL (1 × 10^9^ CFU/mL) F4ac or F4acΔ*faeG* bacterial suspension in DMEM for 1 h, respectively. The normal DMEM is control. After that, cells were harvested in Full moon lysate supplemented with protease and phosphatase inhibitors, and lysis beads were used to ensure complete cell lysis during the vortex process. 50 μg protein sample was diluted in 75 μL labeling buffer and treated with 3 μL Biotin/DMF (10 μg/μL) for 2 h at room temperature. 35 μl of stop reagent was added into the mixture and incubated for another 30 min, and then the resulting biotin-labeled proteins were used for chip hybridization.

The PEX100 chip were mounted in the coupling chamber and flooded with protein coupling mix (the biotin-labeled protein diluted with 3% skimmed milk powder in 6 mL coupling reagent) for 2–3 h at room temperature. After following the washing procedures between each treatment, the chip was incubated with 0.1% Cy3-streptavidin solution for 20 min and processed to Shanghai Ouyi Biomedical Technology Co., Ltd (Shanghai, China) for chip scanning and analysis. The raw data were read by GenePix™ Pro 6.0 software and the extent of protein phosphorylation was calculated by a ratio of the phosphorylated and non-phosphorylated values. The phosphoproteins showed a significant change (ratio ≥ 1.5 or ≤ 0.667, and P-value < 0.05) were included for further study.

### Quantitative real-time PCR analysis

1 × 10^9^ CFUs F4ac or F4acΔ*faeG* bacteria were incubated with IPEC-J2 cell monolayer in a 6-well culture plate (NEST, Shanghai, China) for 1 h at 37 °C, then rinsed the well with PBS three times. Total RNA was extracted from infected and non-infected IPEC-J2 cells using TRIzol (TianGen, Beijing, China), respectively (Duan et al. 2013). The resulting cDNA was synthesized from 1 μg of total RNA using PrimeScript™ 1st strand cDNA Synthesis Kit (Takara Bio, Tokyo, Japan). qRT-PCR reactions were performed in triplicate using UltraSYBR Mixture (Low ROX, CWBIOtech, China) to detect the change of protein expression with different stimuli, and the specific primers used in this experiment listed in Additional file 1: Table S1. All data were normalized to the endogenous reference gene GAPDH and analyzed using the 2^−△△CT^ method (Bustin et al. 2009).

### ELISA analysis

Serum of piglets were collected and the change of d-lactate (d-LA) and diamine oxidase (DAO) concentrations between different groups were detected by Enzyme-linked immunosorbent assay (ELISA) according to the manufacturer’s protocol (Colorful-Gene Biotech, Wuhan, China). After 1-h incubation, the cell-free supernatant from either non-infected and 1 × 10^9^ CFU/mL F4ac or F4ac*ΔfaeG* infected group, 1 μg/mL FaeG protein and LPS treated group were harvested, and the concentrations of caspase-3 (Colorful-Gene Biotech, Wuhan, China), enzymatic activities of caspase-3, -8 and -9 (Beyotime, Beijing, China) were detected by ELISA as well.

### Statistical analyses

The fold change of protein expression was compared among the different stimuli and assayed by quantitative RT-PCR and ELISA assay. The relative value between different groups were analyzed with a Student’s t-test or one-way ANOVA analysis using GraphPad Prism^®^ 8.0 software (GraphPad Prism Inc., CA, USA) and SPSS 16.0 software (SPSS Inc., USA). A p-value of < 0.05 was regarded as significant (*), < 0.01 and < 0.001 were marked as ** and ***, respectively.

## Results

### Deletion of FaeG weaken the F4ac induced intestinal mucosa damage in piglets.

To analyze the change of intestinal barrier function in piglets with F4ac or F4acΔ*faeG* infections, we performed an animal experiment using F4ac receptor-positive piglets. During this process, the weight of piglets in the control group increases steadily, while piglets in F4ac infected group lost weight due to severe diarrhea (Additional file 1: Fig S1). Upon infection, food intake of the infected piglet gradually decreased along with a rough coat, depressed spirit, and continually diarrhea. From the second day post-infection, the piglets in F4ac infected group were mostly dirtied with feces on their hindquarters. After the piglets exposed to CO_2_, the abdominal cavity is opened to observe the pathological changes in the piglet with different treatments. Intestinal edema, mesenteric hemorrhage, congested blood vessel wall, and swollen mesenteric lymph nodes were present in F4ac infected piglets, while there was a mild symptom observed in F4acΔ*faeG* infected group.

**Fig. 1:**
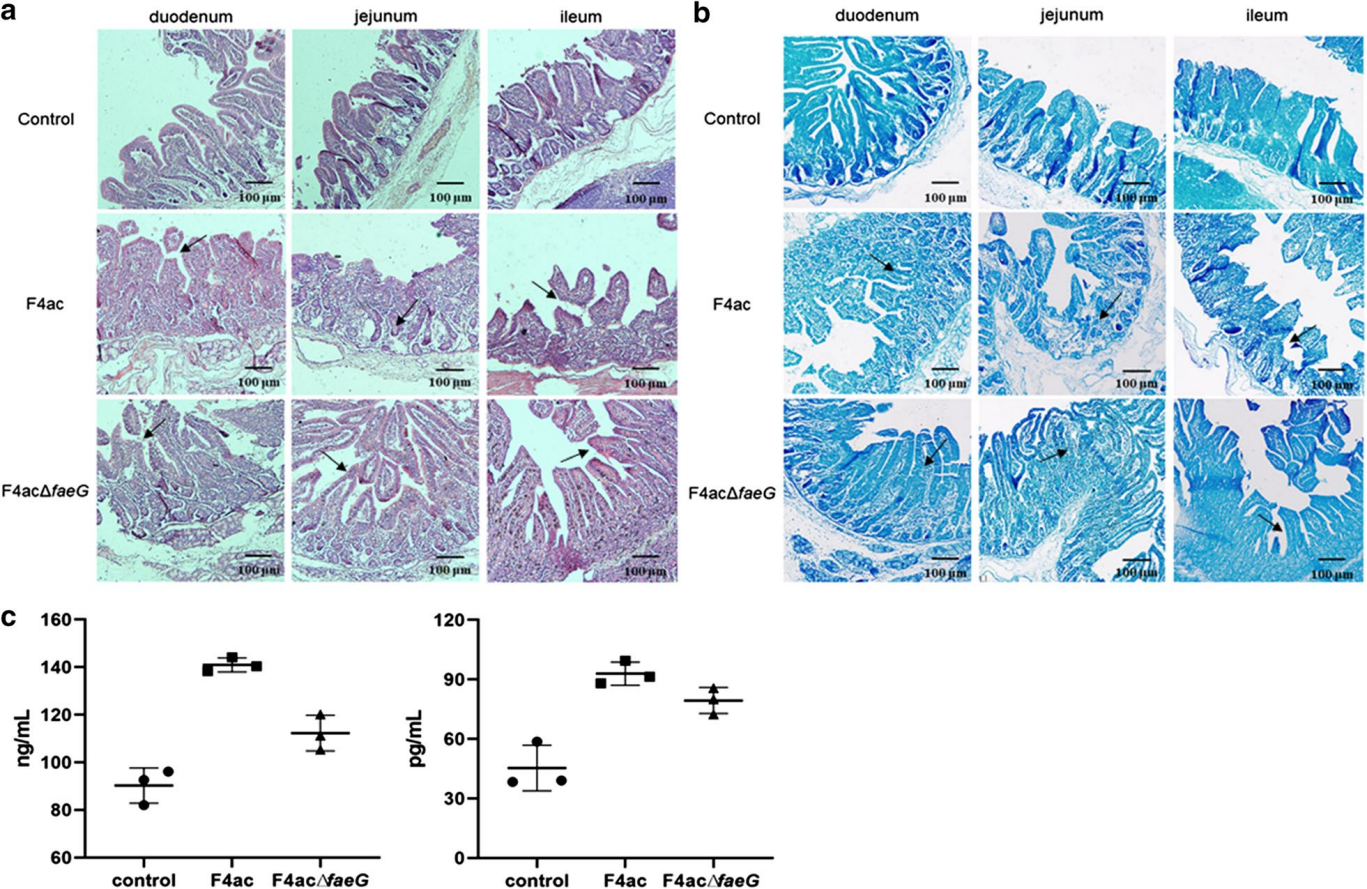
Effect of FaeG deletion on intestinal mucosa morphology and barrier function in piglets. **a** Representative H&E staining image of the small intestine (duodenum, jejunum and ileum). **b** Methylene blue staining results. **c** Serum levels of d-LA (left) and DAO (right) to evalutae the intestinal permeability, *p < 0.05, **p < 0.01

Meanwhile, the duodenum, jejunum, and ileum segments were harvested for further H&E and methylene blue staining. The intestinal mucosa from the control group showed intact structure with neatly and tightly arranged villi, clear and well-organized intestinal wall, compared with the control group, F4acΔ*faeG* infected group exhibited a fractured intestinal mucosa layer in duodenum, as well as a much higher remained intact villi in jejunum and ileum (Fig. 1a). In contrast, in F4ac infected group, the intestinal mucosa layer was severely damaged, the intestinal villi markedly atrophied and shortened in length, and even necrotic and exfoliated in some intestinal tract, along with atrophy occurs in the intestinal glands and congestion in the blood vessel of the intestinal wall.

The result of methylene blue staining confirmed that the F4ac infection leading to reduced methylene blue absorption, while the intestinal epithelial mucosa of both non-infected and F4acΔ*faeG* infected group were stained blue, with villi uniform in length and arranged in an orderly manner (Fig. 1b). Moreover, in order to evaluate intestinal permeability, both d-LA and DAO concentrations were detected in the serum of piglets. Moreover, following the results above, the serum levels of d-LA and DAO increased significantly upon F4ac infection, while they were markedly decreased in F4acΔ*faeG* infected group (Fig. 1c), suggesting that deletion of FaeG remarkably alters F4ac-induced impairment of the intestinal mucosa morphology and barrier function.

### The *faeG* deletion alleviated the F4ac promoted apoptosis in the intestinal epithelial cells.

**Fig. 2:**
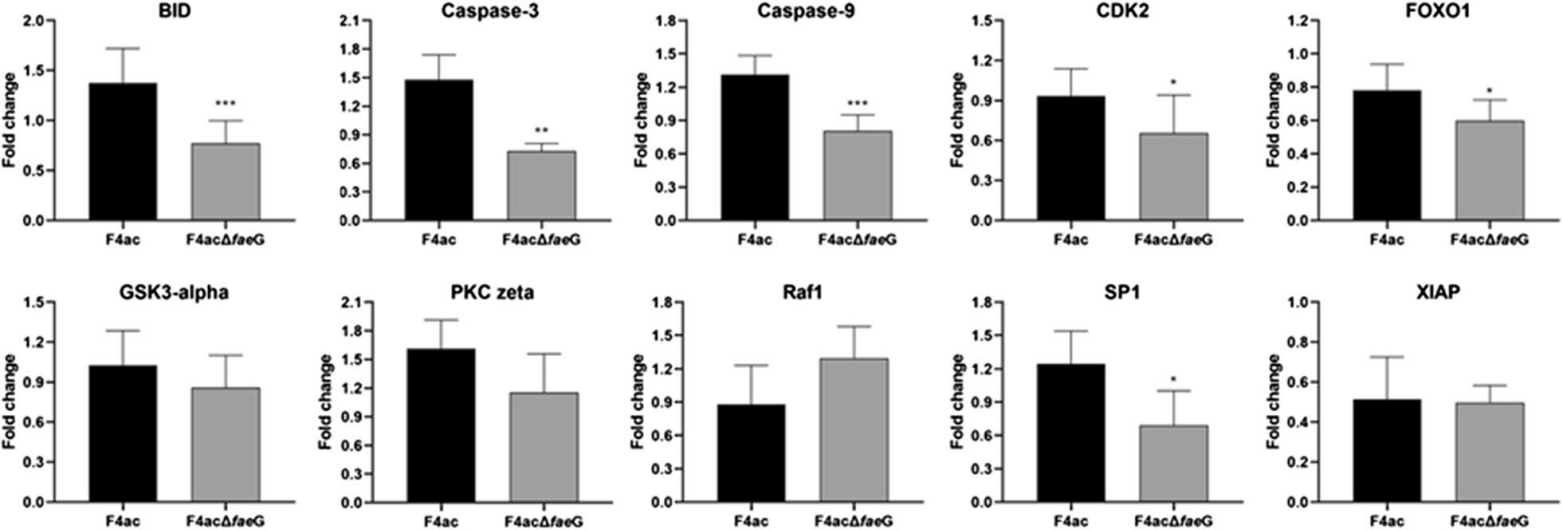
The change of protein expression with different treatments in IPEC-J2 cells. Data are presented as mean ± standard deviations of three independent experiments and normalized to gapdh expression. The asterisk indicates a statistically significant differences, *p < 0.05, **p < 0.01

To find out whether the *faeG* deletion has an effect on the F4ac induced apoptosis in intestinal epithelial cells, we conducted the Phospho Explorer antibody microarray PEX100 analysis to compare changes on 584 phosphorylation sites of 452 key proteins between F4ac and F4acΔ*faeG* infected IPEC-J2 cells and screened out thirty-three proteins that exhibited significant changes in phosphorylation levels. Compared with F4ac infected group, there are 11 proteins phosphorylation upregulated and 22 proteins that were significantly downregulated with the *faeG* deletion mutants’ treatments. These proteins are involved in different signal transductions and diverse cellular functions, and most of them are reported to be closely associated with cell apoptosis. As shown in Table 1, 8 proteins belong to the intrinsic apoptosis pathway, and 4 proteins mainly sense extrinsic signals to undergo apoptosis, while BH3-interacting domain death agonist (BID), caspase-3, -9 and other four proteins participate in both intrinsic and extrinsic apoptosis signaling. qRT-PCR was used to validate the above protein expression change, the mRNA expression of BID, caspase-3, -9, CDK2, FOXO1, and SP1 were decreased in F4acΔ*faeG* infected IPEC-J2 cells compared to that in F4ac treatment (Fig. 2), which is consistent with the results of the antibody microarray analysis.

**Table 1:** Phospho/Unphospho Ratio change in F4ac∆*faeG* vs F4ac infected cells

Phosphorylation site	Gene symbol	F4ac∆*faeG* Phospho/Unphospho ratio (mean ± SD)	F4ac Phospho/Unphospho ratio (mean ± SD)	Fold change (F4ac∆*faeG/*F4ac)	Apoptosis process or type
Abl1 (Phospho-Tyr204)	ABL1	0.217 ± 0.017	0.114 ± 0.016	1.90	Intrinsic (or mitochondrial)
ASK1 (Phospho-Ser83)	MAP3K5	1.347 ± 0.248	0.852 ± 0.233	1.58	Intrinsic (or mitochondrial)
XIAP (Phospho-Ser87)	XIAP	0.563 ± 0.013	0.919 ± 0.050	0.61	Intrinsic
FKHR (Phospho-Ser256)	FOXO1	1.039 ± 0.075	2.172 ± 1.433	0.48	Intrinsic or extrinsic
Cyclin D1 (Phospho-Thr286)	CCND1	0.213 ± 0.101	0.411 ± 0.263	0.52	Intrinsic
p44/42 MAPK (Phospho-Tyr204)	MAPK3	1.102 ± 0.003	1.775 ± 0.722	0.62	Not specific
BID (Phospho-Ser78)	BID	0.418 ± 0.233	0.673 ± 0.093	0.62	Intrinsic or extrinsic
Caspase 3 (Phospho-Ser150)	CASP3	0.888 ± 0.019	1.352 ± 0.037	0.66	Intrinsic or extrinsic
Caspase 9 (Phospho-Tyr153)	CASP9	0.194 ± 0.079	0.333 ± 0.088	0.58	Intrinsic or extrinsic
CDK2 (Phospho-Thr160)	CDK2	1.087 ± 0.017	2.234 ± 0.078	0.49	DNA damage (related to FOXO1)
Cortactin (Phospho-Tyr421)	CTTN	1.217 ± 0.009	5.418 ± 0.196	0.22	Extrinsic
GSK3 alpha (Phospho-Ser21)	GSK3A	1.147 ± 0.060	1.955 ± 0.797	0.59	Intrinsic or extrinsic
NFkB-p100/p52 (Phospho-Ser869)	RELA	1.761 ± 0.696	0.955 ± 0.033	1.84	Not specific
Raf1 (Phospho-Ser259)	RAF1	4.060 ± 0.444	2.561 ± 2.560	1.59	Intrinsic (related to ASK1)
STAT3 (Phospho-Tyr705)	STAT3	0.858 ± 0.017	1.307 ± 0.566	0.66	Intrinsic or extrinsic
NFkB-p65 (Phospho-Thr435)	RELA	2.020 ± 1.050	3.192 ± 0.443	0.63	Not specific
SP1 (Phospho-Thr739)	SP1	1.082 ± 0.052	3.159 ± 2.783	0.34	Not specific
LYN (Phospho-Tyr507)	LYN	0.134 ± 0.064	0.242 ± 0.035	0.55	Not specific
HSP27 (Phospho-Ser82)	HSPB1	1.344 ± 0.084	2.209 ± 0.424	0.61	Intrinsic
Ezrin (Phospho-Thr566)	EZR	0.514 ± 0.269	0.293 ± 0.004	1.75	Not specific (related to XIAP)
GRB10 (Phospho-Tyr67)	GRB10	1.093 ± 0.006	0.712 ± 0.239	1.53	Not specific (related to Bim)
Synuclein alpha (Phospho-Tyr125)	SNCA	0.993 ± 0.030	2.209 ± 1.905	0.45	Intrinsic
VEGFR2 (Phospho-Tyr1175)	KDR	1.034 ± 0.097	1.924 ± 1.630	0.54	Intrinsic
PKC zeta (Phospho-Thr410)	PRKCZ	0.947 ± 0.016	2.130 ± 1.783	0.44	Extrinsic (related to Fas)
Estrogen Receptor-alpha (Phospho-Ser104)	ESR1	2.412 ± 0.150	0.728 ± 0.285	3.31	Extrinsic
Estrogen Receptor-alpha (Phospho-Ser167)	ESR1	1.159 ± 0.015	2.406 ± 1.570	0.48	Extrinsic
CaMK2A (Phospho-Thr286)	CAMK2A	0.318 ± 0.015	0.206 ± 0.013	1.54	Ca^2+^-dependent apoptosis
eEF2K (Phospho-Ser366)	EEF2K	1.011 ± 0.005	0.654 ± 0.183	1.54	Intrinsic or extrinsic
Ras-GRF1 (Phospho-Ser916)	RASGRF1	0.609 ± 0.071	0.396 ± 0.007	1.54	Not specific

**Fig. 3:**
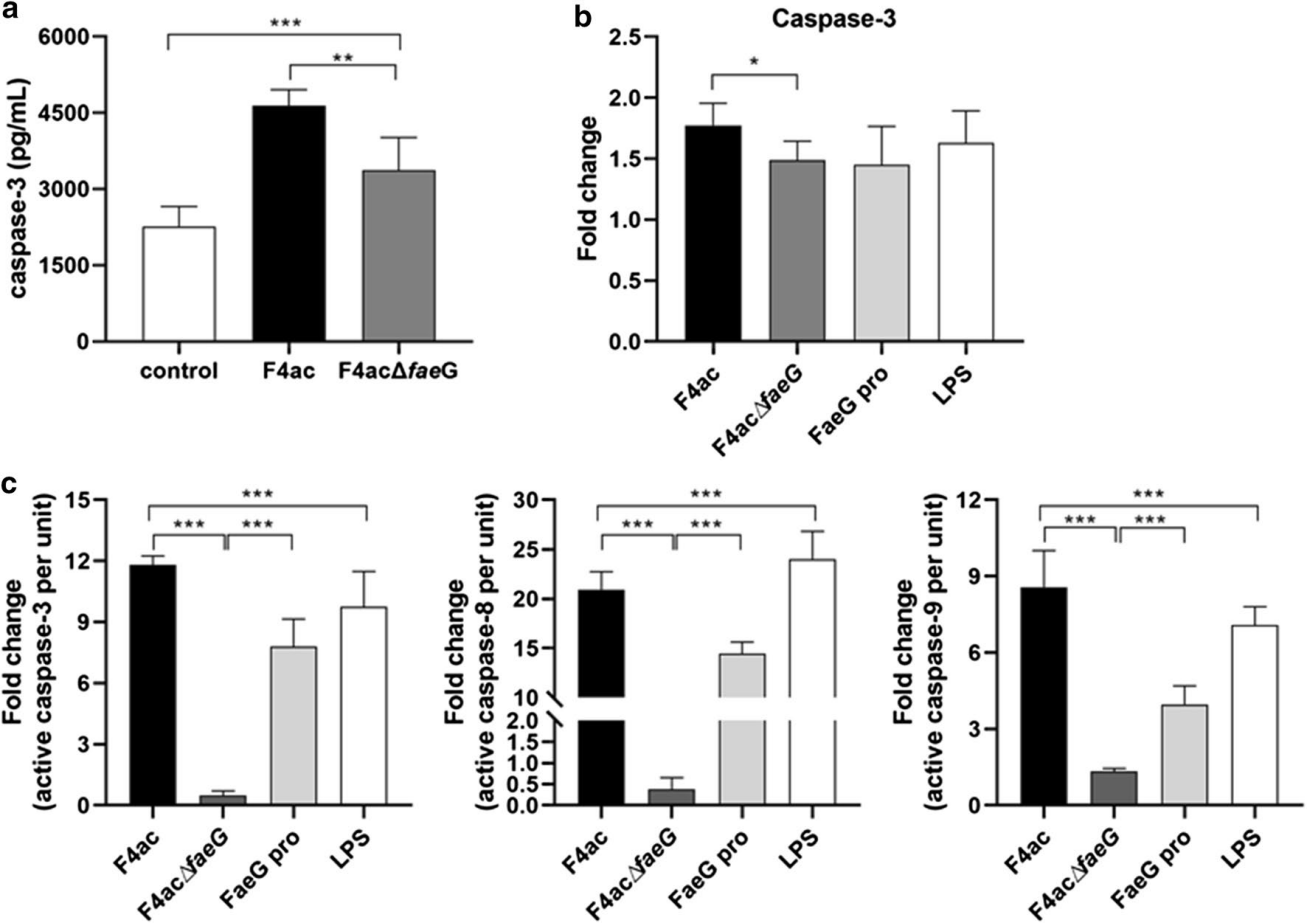
The secretion and enzymatic activities changes of different effector caspases in IPEC-J2 cells with or without bacterial infections. **a** Protein expression of caspase-3 in the serum of piglets. **b** Protein expression of caspase-3 in the bacterial infected IPEC-J2 cells. C. Enzymatic activities of active caspase-3, -8 and -9. The control group without ETEC infection was normalized to 100%. All experiments were repeated three times and data are expressed as mean ± standard deviations (*p < 0.05, **p < 0.01)

Compared with F4ac *E. coli*, F4acΔ*faeG* treatments caused a reduction of caspase-3 expression in both the serum of piglets and the bacterial infected IPEC-J2 cells (Fig. 3a, b). After that, enzymatic activities of caspase-3, -8 and -9 were measured in IPEC-J2 cells from both non-infected and bacterial infected groups (Fig. 3c), and the results proved that the *faeG* deletion leads to a significant decrease in the activation of these effector caspases.

## Discussion

As the initial step for ETEC F4 infection, bacterial attachment to IEC and the interaction between fimbriae and the specific receptor of the host are both crucial for the development of disease. FaeG is among all F4 *fae* operon encoded subunits the major one that involved in fimbrial biosynthesis with other minor subunits, including FaeC, FaeD, FaeE, FaeF, FaeH, etc. In previous study, we found a significant reduction in F4ac∆*faeG* mutants’ adherence to IPEC-J2 and the intestinal brush border cells compared with the parent strains (Xia et al. 2015a). Thus, FaeG is the major subunit of F4ac fimbriae and acts as the most prominent part for F4ac *E. coli* adherence.

As we know, F4ac infections mostly occur in the piglets with the presence of F4ac receptors, and porcine aminopeptidase N (APN) is a newly found F4ac fimbrial receptor in our and other labs’ previous studies (Melkebeek et al. 2012; Xia et al. 2018a). In the later experiment, we proved that the amino acids 149–161, 176–188, and 200–218 are the determinant epitopes for F4ac FaeG to interact with APN directly, and further determined N209 and L212 are the critical sites of F4ac FaeG in binding to the jejunum of piglets (Xia et al. 2018a, 2016). That is to say, FaeG is the target for receptor recognition and can control receptor-mediated binding capacity as well.

Cell apoptosis is a component of natural intestinal epithelial turnover and is involved in regulating intestinal tolerance and homeostasis (Blander 2018; Delgado et al. 2016). Both the intrinsic and the extrinsic pathway of apoptosis have been reported to ultimately lead to the activation of downstream effector caspases, whereas excessive apoptosis in IEC results in increased mucosal permeability and mucosal barrier dysfunction that triggers inflammation and diarrhea (Edelblum et al. 2006; Günther et al. 2013). We first performed the animal infection experiment using the wild type F4ac and F4ac∆*faeG* strains; both the slight change of the intestinal mucosa morphology and the intestinal permeability in F4ac∆*faeG* infected piglets confirmed that the *faeG* deletion attenuates the F4ac-induced impairment of intestinal barrier structure.

After that, we screened out 29 proteins closely related to apoptosis in cells using antibody microarray analysis. The decrease of caspase-3, -9, BID mRNA expression in F4ac∆*faeG* infected cells are in accordance with the change of their phosphorylation ratio. BID is known as the bridging element between intrinsic (or mitochondria-dependent) and extrinsic apoptosis pathway, and the cleavage of BID is active caspase-8 mediated. Besides, it was reported that the activation of caspase-8 is closely related to F4-promoted IEC apoptosis in piglets (Kantari and Walczak 2011; Xia et al. 2018b, 2019). In this regard, we further detected the enzymatic activities of caspase-3, -9 and -8, and confirmed that the *faeG* deletion significantly changed cell apoptosis that occurs upon F4ac infection.

Meanwhile, we detected the enzymatic activities of these caspases in F4ac∆*faeG/pfaeG* (the complemented strain of F4ac∆*faeG*) infected IPEC-J2 cells (data not shown), and found that F4ac∆*faeG/pfaeG* triggered a similar activation of caspase-3, -9 and -8 like F4ac ETEC and there is no significant difference between them. In order to understand the effect of FaeG on cell apoptosis, we used 1 μg FaeG protein to incubate with IPEC-J2 cells and detected the change of enzymatic activities of these caspases, while 1 μg LPS used as the positive control in this test. As shown above, FaeG protein, as an apoptotic inducer, also has the ability to trigger the activation of caspase-3, -9 and -8. Therefore, FaeG affects cell apoptosis, and the *faeG* deletion alleviates ETEC F4ac-induced apoptosis in the intestine, but the molecular and cellular mechanisms remain to be elucidated.

## Supplementary Information

**Additional file 1:** **Table S1.** The specific primers used in this study. **Figure S1.** The change of weight in piglets with different treatments. Data are presented as mean ± standard deviations of three piglets and a one-way analysis of variance (ANOVA) followed by Duncan multiple range test were measured by SPSS.

## Data Availability

The datasets analyzed during the current study are available from the corresponding author on reasonable request.

## Abbreviations

APN: Aminopeptidase N
BID: BH3-interacting domain death agonist
ETEC: Enterotoxigenic *Escherichia coli*
ELISA: Enzyme-linked immunosorbent assay
DAO: Diamine oxidase
d-LA: d-lactate
FBS: Fetal bovine serum
H&E staining: Hematoxylin and eosin staining
IEC: Intestinal epithelial cell
LB: Luria Bertani
