# Supplementary material for: Deletion of FaeG alleviated Enterotoxigenic Escherichia coli F4ac-induced apoptosis in the intestine
Source: AMB Express. 2021 Mar 18;11:44. doi: 10.1186/s13568-021-01201-z (PMC7973317; doi:10.1186/s13568-021-01201-z)

Journal name:

AMB express

Manuscript Title:

Deletion of FaeG alleviated Enterotoxigenic *Escherichia coli* F4ac-induced apoptosis in the intestine

The name(s) of the author(s):

Pengpeng Xia[1,2,3Ξ], Yunping Wu[1,2,3], Siqi Lian[1,2,3], Guomei Quan[1,2,3], Yiting Wang[1,2,3], Guoqiang Zhu[1,2,3Ξ]

The affiliation(s) and address(es) of the author(s):

[1]College of Veterinary Medicine (Institute of comparative medicine), Yangzhou University, Yangzhou 225009, China

[2]Jiangsu Co-innovation Center for Prevention and Control of Important Animal Infectious Diseases and Zoonoses, Yangzhou 225009, China

[3]Joint International Research Laboratory of Agriculture and Agri-Product Safety of Ministry of Education of China, Yangzhou University, Yangzhou 225009, China

The e-mail address, telephone and fax numbers of the corresponding author

Co-Corresponding authors:

1. Pengpeng Xia, College of Veterinary Medicine (Institute of comparative medicine), Yangzhou University, 12th East Wenhui Road, Yangzhou 225009, China.

Tel: (0086)-514-87979033, Fax: (0086)-514-87311374, E-mail: ppxia@yzu.edu.cn

2. Guoqiang Zhu, College of Veterinary Medicine, Yangzhou University, 12th East Wenhui Road, Yangzhou 225009, China.

Tel: (0086)-514-87972590, Fax: (0086)-514-87311374, E-mail: yzgqzhu@yzu.edu.cn

Table S1. The specific primers used in this study.

Primer	Sequence (5’-3’)
GAPDH-F	CCTGCACCACCAACTGCT
GAPDH-R	CACAGTCTTCTGGGTGGC
BID-F	CCTGCTTGTGCTGACTATGC
BID-R	TAGGTGAGGAGGTTCTGGT
Caspase-3-F	GAGGCACAGAATTGGACT
Caspase-3-R	TTTCAGCGCTGCACAAA
Caspase-9-F	CTTCTGCCATGAGTCGGG
Caspase-9-R	CCAAAGCCTGGACCATTT
CDK2-F	ACAAGGTGACGGGAGAAG
CDK2-R	AGATCCTGGTGCAGAAACT
Cortactin-F	CAAGTTTGGTGTCCAGAT
Cortactin-R	ATCCATCCGATCCTTCTGC
Raf1-F	TCTACTCCTACGGCATTGT
Raf1-R	GTGTTGGAGCAGCTCGAT
SP1-F	ACCATGAGCGACCAAGAT
SP1-R	GTGTGGCTGTGAGGTCAA
XIAP-F	GTGCATGGTCAGAACACA
XIAP-R	GCCATGGCTGGATTTCTT

Figure S1. The change of weight in piglets with different treatments. Data are presented as mean ± standard deviations of three piglets and a one-way analysis of variance (ANOVA) followed by Duncan multiple range test were measured by SPSS.

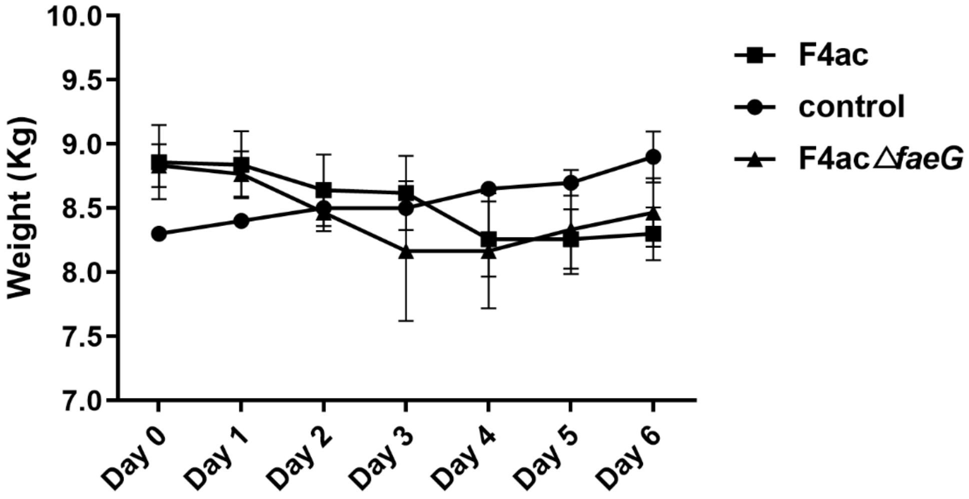

Supplement: Supplementary file 1 — Additional file 1: Table S1. The specific primers used in this study. Figure S1. The change of weight in piglets with different treatments. Data are presented as mean ± standard deviations of three piglets and a one-way analysis of variance (ANOVA) followed by Duncan multiple range test were measured by SPSS. [file 13568_2021_1201_MOESM1_ESM.pdf]
